# 2-Oxo-2*H*-chromen-4-yl 4-meth­oxy­benzoate

**DOI:** 10.1107/S1600536812047666

**Published:** 2012-11-24

**Authors:** Akoun Abou, Abdoulaye Djandé, Grégoire Danger, Adama Saba, Rita Kakou-Yao

**Affiliations:** a Laboratoire de Cristallographie et Physique Moléculaire, UFR SSMT, Université de Cocody, 22 BP 582 Abidjan 22, Côte d’Ivoire; b Laboratoire de Chimie Bio-organique et de Phytochimie, Université de Ouagadougou, 03 BP 7021 Ouagadougou 03, Burkina Faso; c Laboratoire de Physique des Interactions Ioniques et Moléculaires, Equipe-Spectrométries et Dynamique Moléculaire, Centre Saint Jérôme, Université de Provence, 13397 Marseille, France

## Abstract

In the title mol­ecule, C_17_H_12_O_5_, the chromen-2-one ring and the 4-meth­oxy­benzoate side chain are inclined to one another at a dihedral angle of 69.82 (9)°. The crystal structure features parallel sheets of centrosymmetric *R*_2_^2^(6) dimers joined by a *C*(7) chain, resulting in centrosymetric tetra­mers of hydrogen-bonded mol­ecules with graph-set motif *R*_4_^4^(40). These centrosymetric tetra­mers are connected by a pair of hydrogen bonds described by an *R*_2_^2^(8) ring motif and a *C*(7) chain *via* C—H⋯O inter­actions. In the structure, there are also π–π stacking inter­actions between chromene benzene and the six-membered heterocyclic rings [centroid–centroid distance = 3.691 (2) Å] and weak C=O⋯π inter­actions [O⋯(ring centroid) distance = 3.357 (3) Å].

## Related literature

For the biological activity of coumarin derivatives, see: Basanagouda *et al.* (2009 ▶); Vukovic *et al.* (2010 ▶); Emmanuel-Giota *et al.* (2001 ▶); Marchenko *et al.* (2006 ▶). For hydrogen-bond motifs, see: Bernstein *et al.* (1995 ▶). For π–π stacking inter­actions, see: Janiak (2000 ▶).
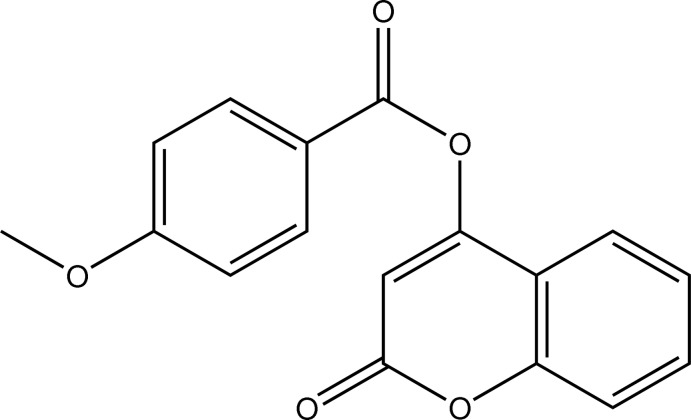


## Experimental

### 

#### Crystal data

C_17_H_12_O_5_
*M* *_r_* = 296.27
Triclinic, 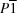

*a* = 4.371 (1) Å
*b* = 10.535 (4) Å
*c* = 15.193 (2) Å
α = 85.218 (3)°
β = 83.688 (2)°
γ = 81.893 (1)°
*V* = 686.8 (3) Å^3^
*Z* = 2
Mo *K*α radiation
μ = 0.11 mm^−1^
*T* = 298 K
0.25 × 0.15 × 0.04 mm

#### Data collection

Nonius KappaCCD diffractometer
5683 measured reflections
2731 independent reflections
1540 reflections with *I* > 2σ(*I*)
*R*_int_ = 0.055

#### Refinement

*R*[*F* ^2^ > 2σ(*F* ^2^)] = 0.066
*wR*(*F* ^2^) = 0.163
*S* = 1.11
2731 reflections
200 parameters
H-atom parameters constrained
Δρ_max_ = 0.17 e Å^−3^
Δρ_min_ = −0.23 e Å^−3^

### 

Data collection: *COLLECT* (Hooft, 1998 ▶); cell refinement: *DENZO*/*SCALEPACK* (Otwinowski & Minor, 1997 ▶); data reduction: *DENZO*/*SCALEPACK*; program(s) used to solve structure: *SIR2004* (Burla *et al.*, 2005 ▶); program(s) used to refine structure: *SHELXL97* (Sheldrick, 2008 ▶); molecular graphics: *PLATON* (Spek, 2009 ▶); software used to prepare material for publication: *SHELXL97*, *publCIF* (Westrip, 2010 ▶) and *WinGX* (Farrugia, 2012 ▶).

## Supplementary Material

Crystal structure: contains datablock(s) I, global. DOI: 10.1107/S1600536812047666/zs2245sup1.cif

Structure factors: contains datablock(s) I. DOI: 10.1107/S1600536812047666/zs2245Isup2.hkl

Supplementary material file. DOI: 10.1107/S1600536812047666/zs2245Isup3.cml

Additional supplementary materials:  crystallographic information; 3D view; checkCIF report

## Figures and Tables

**Table 1:** Hydrogen-bond geometry (Å, °)

*D*—H⋯*A*	*D*—H	H⋯*A*	*D*⋯*A*	*D*—H⋯*A*
C8—H8⋯O2^i^	0.93	2.48	3.407 (4)	173
C2—H2⋯O4^ii^	0.93	2.49	3.340 (4)	151
C17—H17*B*⋯O5^iii^	0.96	2.59	3.461 (4)	151

Symmetry codes: (i) 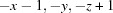; (ii) 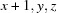; (iii) 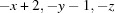.

**Fig. 1.**
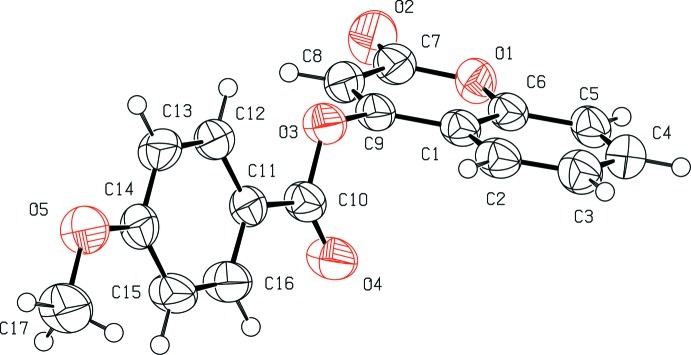
The molecular structure of the title compound, showing displacement ellipsoids at the 50% probability level. H atoms are shown as spheres of arbitrary radius.

**Fig. 2.**
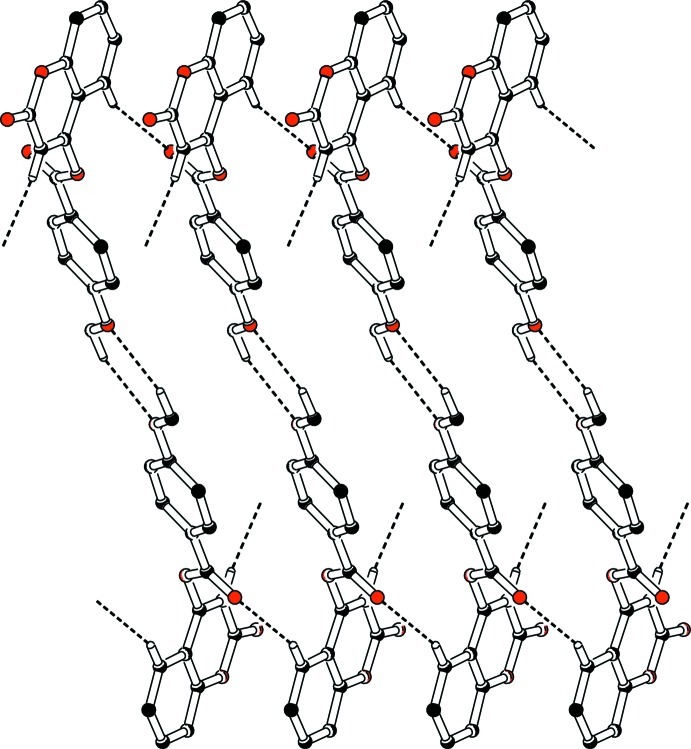
The crystal packing, viewed down the c axis, showing parallel sheets of centrosymmetric R22(6) dimers linked by an infinite C(7) chain to form a centrosymetric R44(40) tetramers. The dashed lines indicate hydrogen bonds. H atoms not involved in hydrogen bonding have been omitted for clarity.

**Fig. 3.**
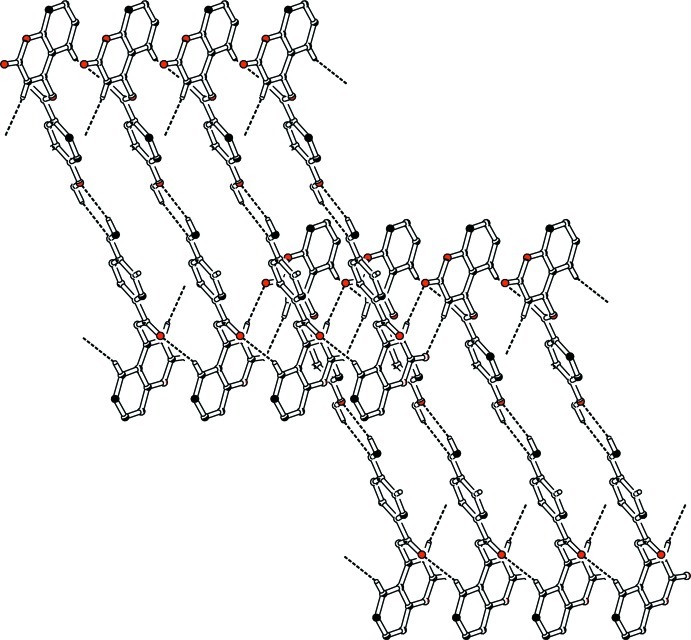
The crystal packing, viewed down the c axis, showing the supramolecular aggregation formed by the propagation of the centrosymetric R44(40) tetramers via C—H···O hydrogen bonds. The dashed lines indicate hydrogen bonds. H atoms not involved in hydrogen bonding have been omitted.

**Fig. 4.**
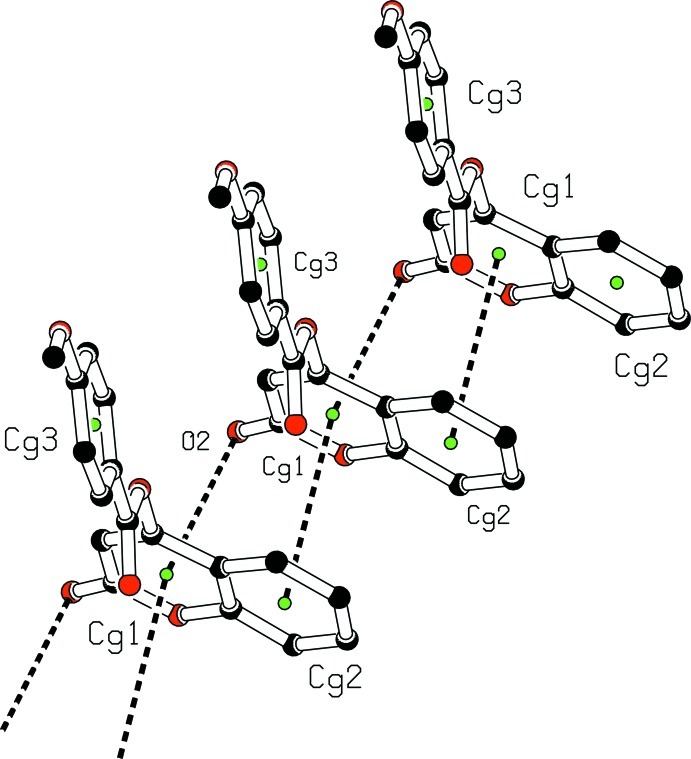
A view of the crystal packing, showing C═O···π and π–π stacking interactions (dashed lines). The green dots are centroids of rings. H atoms have been omitted.

## supplementary crystallographic information

### Comment

Coumarin constitutes one of the major classes of naturally occurring compounds and interest in its chemistry continues unabated because of its usefulness as a biologically active agent. It also represents the core structure of several molecules of pharmaceutical importance. Coumarin and its derivatives have been reported to serve as anti-bacterial (Basanagouda *et al.*, 2009), anti-oxidant (Vukovic *et al.*, 2010), anti-inflammatory (Emmanuel-Giota *et al.*, 2001) and anti-tumour agents (Marchenko, *et al.*, 2006). Therefore, the synthesis of new coumarin derivatives is of considerable interest. In order to study the influence of new substituents on the activity of the coumarin derivatives, the title compound, the ester C_17_H_12_O_5_ has been synthesized and its molecular and crystal structure is reported herein.

In the title compound (Fig. 1), the side chain is tilted with respect to the chromen-2-one ring with torsion angles C1—C9—O3—C10 = -76.3 (4)° and C8—C9—O3—C10 = 107.7 (3)°. The dihedral angle between the chromene ring and the side chain is 69.82 (9) °.

In the crystal structure, weak intermolecular C—H···O hydrogen bonds (Table 1) generate hydrogen-bonding motifs ranging from a chain to various rings. Indeed, in the methoxy group, an H atom of the methyl group (H17*B*) bonds to the oxygen atom of the same group on a neighbouring molecule (related by an inversion center) to form parallel sheets of centrosymetric dimers [graph set *R*_2_^2^(6) (Bernstein *et al.*, 1995)]. Also, a hydrogen of the chromene-benzene ring (H2) bonds to the oxygen atom of the carbonyl group of the side chain of a neighbouring molecule to form an infinite chain [graph set *C*(7)]. The combination of the *C*(7) chain and the *R*_2_^2^(6) dimers results in a ring of hydrogen-bonded molecules described by the graph set *R*_4_^4^(40) (Fig. 2). Further, the hydrogen of the six-membered heterocyclic ring bonds to the oxygen atom of the carbonyl group of the chromen-2-one moiety of an inversion-related neighbouring molecule to form a pair of hydrogen bonds [graph set *R*_2_^2^(8)]. The latter hydrogen bonds and the *C*(7) chain connect the *R*_4_^4^(40) centrosymmetric tetramers, resulting in a supramolecular aggregation (Fig. 3) which is further consolidated by weak C═ O···π interactions [O2···*Cg*1 (*x* - 1, *y*, *z*) = 3.357 (3) Å], where *Cg*1 is the centroid of the six-membered O containing ring, and π–π stacking between the chromene-benzene C1—C6 and the six-membered heterocyclic rings; in the latter, the centroid···centroid distance, [*Cg*2···*Cg*1 (*x* + 1, *y*, *z*) or *Cg*1···*Cg*2 (*x* - 1, *y*, *z*) = 3.691 (2) Å], is less than 3.8 Å, the maximum regarded as relevant for π–π interactions (Janiak, 2000) (Fig. 4).

### Experimental

To a solution of 4-methoxybenzoyl chloride (40 mmol) in dried tetrahydrofuran (150 ml), was added dried triethylamine (120 mmol) and 4-hydroxycoumarin (40 mmol)in small portions over 30 min. The mixture was then refluxed for 3 h and poured in 300 ml of chloroform. The solution was acidified with dilute hydrochloric acid until the pH was 2–3. The organic layer was extracted, washed with water, dried over MgSO_4_ and the solvent removed. The crude product was recrystallized from chloroform. Colourless crystals of the title compound were obtained in a good yield (84%); m.p. 421–422 K. ^1^H NMR (Bruker TOPSPIN, CDCl_3_, 400 MHz, p.p.m.) δ: 6.63 (s, 1H, H8); 7.43 (d, 1H, H2); 7.33 (t.d, 1H, H3); 7.61 (t.d,1*H*, H4); 7.73 (d, 1H, H5); 8.2 (d, 2H, H12 and H16); 7.05 (d, 2H, H13 and H15); 3.93 (s, 3H, CH_3_). ^13^C NMR (Bruker TOPSPIN, CDCl_3_, 100 MHz, p.p.m.) δ: 162 (C7); 108 (C8); 161 (C9); 127 (C2); 124 (C3); 117 (C4); 126 (C5); 153 (C6); 115 (C1); 165 (C10); 160 (C11); 133 (C12 and C16); 113 (C13 and C15); 120 (C14); 55 (C17).

### Refinement

H atoms were placed in calculated positions [C—H = 0.93 (aromatic) or 0.96 Å (methyl group)] and refined using a riding model approximation with *U*_iso_(H) constrained to 1.2 (aromatic) or 1.5 (methyl) times *U*_eq_ of the respective parent atom. The five reflections (1 - 5 17), (0 - 1 1), (0 0 1), (0 1 0), (1 0 1) were found to have too low intensities, caused by a systematic error, probably by shielding by the beam stop interference. They were omitted from the refinement.

### Crystal data

**Table:**

C_17_H_12_O_5_	*Z* = 2
*M**_r_* = 296.27	*F*(000) = 308
Triclinic, *P*1	*D*_x_ = 1.433 Mg m^−^^3^
Hall symbol: -P 1	Melting point = 421–422 K
*a* = 4.371 (1) Å	Mo *K*α radiation, λ = 0.71073 Å
*b* = 10.535 (4) Å	Cell parameters from 5683 reflections
*c* = 15.193 (2) Å	θ = 2.3–27.0°
α = 85.218 (3)°	µ = 0.11 mm^−^^1^
β = 83.688 (2)°	*T* = 298 K
γ = 81.893 (1)°	Prism, colourless
*V* = 686.8 (3) Å^3^	0.25 × 0.15 × 0.04 mm

### Data collection

**Table:**

Nonius KappaCCD diffractometer	1540 reflections with *I* > 2σ(*I*)
Radiation source: fine-focus sealed tube	*R*_int_ = 0.055
Graphite monochromator	θ_max_ = 27.0°, θ_min_ = 2.3°
φ and ω scans	*h* = 0→5
5683 measured reflections	*k* = −12→13
2731 independent reflections	*l* = −18→19

### Refinement

**Table:**

Refinement on *F*^2^	Primary atom site location: structure-invariant direct methods
Least-squares matrix: full	Secondary atom site location: difference Fourier map
*R*[*F*^2^ > 2σ(*F*^2^)] = 0.066	Hydrogen site location: inferred from neighbouring sites
*wR*(*F*^2^) = 0.163	H-atom parameters constrained
*S* = 1.11	*w* = 1/[σ^2^(*F*_o_^2^) + (0.0324*P*)^2^ + 0.5861*P*] where *P* = (*F*_o_^2^ + 2*F*_c_^2^)/3
2731 reflections	(Δ/σ)_max_ < 0.001
200 parameters	Δρ_max_ = 0.17 e Å^−^^3^
0 restraints	Δρ_min_ = −0.23 e Å^−^^3^
48 constraints	

### Special details

**Table:**

Geometry. All s.u.'s (except the s.u. in the dihedral angle between two l.s. planes) are estimated using the full covariance matrix. The cell s.u.'s are taken into account individually in the estimation of s.u.'s in distances, angles and torsion angles; correlations between s.u.'s in cell parameters are only used when they are defined by crystal symmetry. An approximate (isotropic) treatment of cell s.u.'s is used for estimating s.u.'s involving l.s. planes.
Refinement. Refinement of *F*^2^ against ALL reflections. The weighted *R*-factor *wR* and goodness of fit *S* are based on *F*^2^, conventional *R*-factors *R* are based on *F*, with *F* set to zero for negative *F*^2^. The threshold expression of *F*^2^ > 2σ(*F*^2^) is used only for calculating *R*-factors(gt) *etc*. and is not relevant to the choice of reflections for refinement. *R*-factors based on *F*^2^ are statistically about twice as large as those based on *F*, and *R*- factors based on ALL data will be even larger.

### Fractional atomic coordinates and isotropic or equivalent isotropic displacement parameters (Å^2^)

**Table:**

	*x*	*y*	*z*	*U*_iso_*/*U*_eq_	
O3	0.1598 (5)	0.05340 (19)	0.31179 (13)	0.0542 (6)	
O1	−0.3230 (5)	0.3325 (2)	0.47003 (14)	0.0528 (6)	
O2	−0.5808 (6)	0.1879 (2)	0.54685 (17)	0.0722 (7)	
C11	0.2683 (7)	−0.0573 (3)	0.1811 (2)	0.0447 (7)	
O4	−0.1068 (6)	0.1288 (2)	0.19569 (15)	0.0656 (7)	
O5	0.7433 (6)	−0.3598 (2)	0.04518 (16)	0.0702 (7)	
C5	−0.0670 (8)	0.4966 (3)	0.3926 (2)	0.0525 (8)	
H5	−0.1839	0.5552	0.4297	0.063*	
C9	−0.0098 (7)	0.1490 (3)	0.3629 (2)	0.0459 (8)	
C1	0.0601 (7)	0.2784 (3)	0.3473 (2)	0.0443 (7)	
C6	−0.1068 (7)	0.3679 (3)	0.4027 (2)	0.0455 (8)	
C14	0.5790 (7)	−0.2567 (3)	0.0861 (2)	0.0512 (8)	
C10	0.0876 (8)	0.0505 (3)	0.2267 (2)	0.0500 (8)	
C4	0.1473 (8)	0.5360 (3)	0.3273 (2)	0.0586 (9)	
H4	0.1758	0.6222	0.3199	0.070*	
C7	−0.3849 (8)	0.2081 (3)	0.4861 (2)	0.0532 (8)	
C12	0.4725 (7)	−0.1501 (3)	0.2215 (2)	0.0510 (8)	
H12	0.5054	−0.1455	0.2805	0.061*	
C15	0.3792 (8)	−0.1649 (3)	0.0454 (2)	0.0612 (10)	
H15	0.3468	−0.1691	−0.0138	0.073*	
C8	−0.2172 (8)	0.1147 (3)	0.4278 (2)	0.0520 (8)	
H8	−0.2550	0.0296	0.4359	0.062*	
C13	0.6278 (8)	−0.2498 (3)	0.1738 (2)	0.0565 (9)	
H13	0.7656	−0.3125	0.2008	0.068*	
C16	0.2264 (8)	−0.0658 (3)	0.0939 (2)	0.0618 (10)	
H16	0.0905	−0.0027	0.0665	0.074*	
C3	0.3223 (8)	0.4490 (3)	0.2719 (2)	0.0585 (9)	
H3	0.4692	0.4768	0.2283	0.070*	
C2	0.2795 (7)	0.3217 (3)	0.2813 (2)	0.0525 (8)	
H2	0.3965	0.2639	0.2437	0.063*	
C17	0.7156 (10)	−0.3667 (4)	−0.0473 (2)	0.0784 (12)	
H17A	0.5023	−0.3692	−0.0560	0.118*	
H17B	0.8401	−0.4429	−0.0680	0.118*	
H17C	0.7859	−0.2924	−0.0800	0.118*	

### Atomic displacement parameters (Å^2^)

**Table:**

	*U*^11^	*U*^22^	*U*^33^	*U*^12^	*U*^13^	*U*^23^
O3	0.0695 (15)	0.0459 (12)	0.0453 (13)	0.0091 (10)	−0.0096 (11)	−0.0155 (10)
O1	0.0634 (14)	0.0459 (13)	0.0478 (13)	−0.0031 (10)	0.0020 (11)	−0.0138 (10)
O2	0.0892 (19)	0.0661 (16)	0.0575 (16)	−0.0115 (13)	0.0144 (14)	−0.0096 (12)
C11	0.0515 (19)	0.0393 (17)	0.0429 (18)	−0.0017 (13)	−0.0024 (14)	−0.0103 (14)
O4	0.0724 (16)	0.0624 (15)	0.0601 (15)	0.0189 (12)	−0.0207 (13)	−0.0215 (12)
O5	0.0938 (19)	0.0529 (14)	0.0594 (16)	0.0174 (13)	−0.0090 (13)	−0.0219 (12)
C5	0.060 (2)	0.0451 (19)	0.054 (2)	0.0007 (15)	−0.0131 (17)	−0.0166 (15)
C9	0.057 (2)	0.0391 (17)	0.0418 (18)	0.0060 (14)	−0.0117 (16)	−0.0130 (14)
C1	0.0482 (18)	0.0443 (18)	0.0404 (17)	0.0026 (14)	−0.0096 (14)	−0.0106 (14)
C6	0.0508 (19)	0.0458 (18)	0.0407 (18)	−0.0030 (14)	−0.0080 (15)	−0.0095 (14)
C14	0.060 (2)	0.0394 (17)	0.053 (2)	0.0007 (15)	−0.0023 (16)	−0.0139 (15)
C10	0.055 (2)	0.0488 (19)	0.047 (2)	−0.0023 (16)	−0.0085 (16)	−0.0116 (15)
C4	0.070 (2)	0.047 (2)	0.061 (2)	−0.0107 (17)	−0.0139 (19)	−0.0028 (17)
C7	0.065 (2)	0.049 (2)	0.045 (2)	−0.0026 (16)	−0.0074 (17)	−0.0086 (15)
C12	0.063 (2)	0.0443 (18)	0.0452 (19)	−0.0020 (15)	−0.0084 (16)	−0.0076 (15)
C15	0.077 (2)	0.059 (2)	0.047 (2)	0.0108 (18)	−0.0159 (18)	−0.0186 (17)
C8	0.066 (2)	0.0430 (18)	0.0464 (19)	−0.0012 (15)	−0.0070 (17)	−0.0073 (15)
C13	0.071 (2)	0.0420 (18)	0.053 (2)	0.0105 (16)	−0.0108 (17)	−0.0073 (16)
C16	0.078 (2)	0.054 (2)	0.051 (2)	0.0165 (17)	−0.0171 (18)	−0.0157 (17)
C3	0.065 (2)	0.062 (2)	0.051 (2)	−0.0137 (17)	−0.0056 (17)	−0.0051 (17)
C2	0.056 (2)	0.057 (2)	0.0447 (19)	0.0012 (16)	−0.0072 (16)	−0.0129 (15)
C17	0.103 (3)	0.071 (3)	0.057 (2)	0.015 (2)	−0.006 (2)	−0.029 (2)

### Geometric parameters (Å, º)

**Table:**

O3—C10	1.368 (4)	C14—C15	1.365 (4)
O3—C9	1.400 (3)	C14—C13	1.381 (4)
O1—C7	1.373 (4)	C4—C3	1.387 (5)
O1—C6	1.379 (4)	C4—H4	0.9300
O2—C7	1.214 (4)	C7—C8	1.445 (4)
C11—C16	1.369 (4)	C12—C13	1.381 (4)
C11—C12	1.380 (4)	C12—H12	0.9300
C11—C10	1.467 (4)	C15—C16	1.379 (4)
O4—C10	1.204 (4)	C15—H15	0.9300
O5—C14	1.370 (3)	C8—H8	0.9300
O5—C17	1.433 (4)	C13—H13	0.9300
C5—C4	1.368 (4)	C16—H16	0.9300
C5—C6	1.385 (4)	C3—C2	1.373 (4)
C5—H5	0.9300	C3—H3	0.9300
C9—C8	1.327 (4)	C2—H2	0.9300
C9—C1	1.434 (4)	C17—H17A	0.9600
C1—C6	1.391 (4)	C17—H17B	0.9600
C1—C2	1.403 (4)	C17—H17C	0.9600
			
C10—O3—C9	117.2 (2)	O1—C7—C8	116.9 (3)
C7—O1—C6	122.2 (2)	C11—C12—C13	119.6 (3)
C16—C11—C12	119.0 (3)	C11—C12—H12	120.2
C16—C11—C10	117.4 (3)	C13—C12—H12	120.2
C12—C11—C10	123.6 (3)	C14—C15—C16	118.4 (3)
C14—O5—C17	117.5 (3)	C14—C15—H15	120.8
C4—C5—C6	118.9 (3)	C16—C15—H15	120.8
C4—C5—H5	120.6	C9—C8—C7	120.8 (3)
C6—C5—H5	120.6	C9—C8—H8	119.6
C8—C9—O3	118.5 (3)	C7—C8—H8	119.6
C8—C9—C1	122.3 (3)	C12—C13—C14	120.2 (3)
O3—C9—C1	119.1 (3)	C12—C13—H13	119.9
C6—C1—C2	117.9 (3)	C14—C13—H13	119.9
C6—C1—C9	116.4 (3)	C11—C16—C15	122.1 (3)
C2—C1—C9	125.7 (3)	C11—C16—H16	118.9
O1—C6—C5	116.8 (3)	C15—C16—H16	118.9
O1—C6—C1	121.3 (3)	C2—C3—C4	120.2 (3)
C5—C6—C1	121.9 (3)	C2—C3—H3	119.9
C15—C14—O5	123.9 (3)	C4—C3—H3	119.9
C15—C14—C13	120.6 (3)	C3—C2—C1	120.4 (3)
O5—C14—C13	115.5 (3)	C3—C2—H2	119.8
O4—C10—O3	121.8 (3)	C1—C2—H2	119.8
O4—C10—C11	125.8 (3)	O5—C17—H17A	109.5
O3—C10—C11	112.4 (3)	O5—C17—H17B	109.5
C5—C4—C3	120.8 (3)	H17A—C17—H17B	109.5
C5—C4—H4	119.6	O5—C17—H17C	109.5
C3—C4—H4	119.6	H17A—C17—H17C	109.5
O2—C7—O1	116.6 (3)	H17B—C17—H17C	109.5
O2—C7—C8	126.4 (3)		
			
C10—O3—C9—C8	107.7 (3)	C6—C5—C4—C3	0.0 (5)
C10—O3—C9—C1	−76.3 (4)	C6—O1—C7—O2	−179.7 (3)
C8—C9—C1—C6	−1.4 (4)	C6—O1—C7—C8	−1.3 (4)
O3—C9—C1—C6	−177.3 (3)	C16—C11—C12—C13	−0.6 (5)
C8—C9—C1—C2	178.8 (3)	C10—C11—C12—C13	178.6 (3)
O3—C9—C1—C2	2.9 (5)	O5—C14—C15—C16	179.8 (3)
C7—O1—C6—C5	179.5 (3)	C13—C14—C15—C16	−0.3 (5)
C7—O1—C6—C1	0.1 (4)	O3—C9—C8—C7	176.2 (3)
C4—C5—C6—O1	179.4 (3)	C1—C9—C8—C7	0.3 (5)
C4—C5—C6—C1	−1.3 (5)	O2—C7—C8—C9	179.3 (3)
C2—C1—C6—O1	−179.0 (3)	O1—C7—C8—C9	1.1 (5)
C9—C1—C6—O1	1.2 (4)	C11—C12—C13—C14	0.0 (5)
C2—C1—C6—C5	1.7 (4)	C15—C14—C13—C12	0.5 (5)
C9—C1—C6—C5	−178.1 (3)	O5—C14—C13—C12	−179.7 (3)
C17—O5—C14—C15	3.8 (5)	C12—C11—C16—C15	0.7 (5)
C17—O5—C14—C13	−176.1 (3)	C10—C11—C16—C15	−178.5 (3)
C9—O3—C10—O4	2.2 (5)	C14—C15—C16—C11	−0.3 (6)
C9—O3—C10—C11	−177.0 (3)	C5—C4—C3—C2	0.9 (5)
C16—C11—C10—O4	4.1 (5)	C4—C3—C2—C1	−0.5 (5)
C12—C11—C10—O4	−175.1 (3)	C6—C1—C2—C3	−0.8 (5)
C16—C11—C10—O3	−176.7 (3)	C9—C1—C2—C3	179.0 (3)
C12—C11—C10—O3	4.1 (4)		

### Hydrogen-bond geometry (Å, º)

**Table:**

*D*—H···*A*	*D*—H	H···*A*	*D*···*A*	*D*—H···*A*
C8—H8···O2^i^	0.93	2.48	3.407 (4)	173
C2—H2···O4^ii^	0.93	2.49	3.340 (4)	151
C17—H17*B*···O5^iii^	0.96	2.59	3.461 (4)	151

Symmetry codes: (i) −*x*−1, −*y*, −*z*+1; (ii) *x*+1, *y*, *z*; (iii) −*x*+2, −*y*−1, −*z*.
